# RNAs and Gene Expression Predicting Postoperative Atrial Fibrillation in Cardiac Surgery Patients Undergoing Coronary Artery Bypass Grafting

**DOI:** 10.3390/jcm9041139

**Published:** 2020-04-16

**Authors:** Muhammad Shuja Khan, Kennosuke Yamashita, Vikas Sharma, Ravi Ranjan, Derek James Dosdall

**Affiliations:** 1 Nora Eccles Harrison Cardiovascular Research and Training Institute, The University of Utah, Salt Lake City, UT 84112, USA; m.khan@utah.edu (M.S.K.); kennosuke.atmm3@gmail.com (K.Y.); ravi.ranjan@hsc.utah.edu (R.R.); 2 Division of Cardiovascular Medicine, The University of Utah-Health, Salt Lake City, UT 84132, USA; 3 Division of Cardiothoracic Surgery, The University of Utah-Health, Salt Lake City, UT 84132, USA; vikas.sharma@hsc.utah.edu; 4 Department of Biomedical Engineering, The University of Utah, Salt Lake City, UT 84112, USA

**Keywords:** postoperative atrial fibrillation, biomarkers, coronary artery bypass grafting, miRNA, circRNA, mtDNA, SNPs, atrial fibrillation

## Abstract

Postoperative atrial fibrillation (POAF) is linked with increased morbidity, mortality rate and financial liability. About 20–50% of patients experience POAF after coronary artery bypass graft (CABG) surgery. Numerous review articles and meta-analyses have investigated links between patient clinical risk factors, demographic conditions, and pre-, peri- and post-operative biomarkers to forecast POAF incidence in CABG patients. This narrative review, for the first time, summarize the role of micro-RNAs, circular-RNAs and other gene expressions that have shown experimental evidence to accurately predict the POAF incidence in cardiac surgery patients after CABG. We envisage that identifying specific genomic markers for predicting POAF might be a significant step for the prevention and effective management of this type of post-operative complication and may provide critical perspective into arrhythmogenic substrate responsible for POAF.

## 1. Introduction

Postoperative atrial fibrillation (POAF) occurs in 20–50% of coronary artery bypass graft (CABG) patients during postoperative stay [1]. It has been reported that the incidence of POAF is approximately 50% after combined CABG/valvular procedures, 30% after pure CABG surgery, and 40% following valve replacements or repair [2,3,4]. Although POAF is generally considered to be a transitory condition, it can be life-threatening and is associated with increased complications, morbidity, mortality rate and financial burden [4]. In cardiac patients that experienced an arrhythmia, 70% of them developed POAF before the end of the fourth postoperative day and 94% before the end of the sixth postoperative day [5]. Treatment of POAF is estimated to add an additional $1 billion in health care costs in the US alone [6]. Therefore, identifying patients at an early stage may help to define a population that is more likely to benefit from anti-arrhythmic drug therapy or additional surgical intervention during the open chest procedure and may lead to a substantial reduction in POAF in the highest risk patients [7,8,9]. Reducing the rate of POAF is linked with a decline in the extent of hospital stay and possible cost savings [4].

There have been several algorithms and theories used to forecast the complications of post-CABG surgery, however, current methods are established based-on patients’ demographics and clinical co-morbidities, and preoperative performance status [3,10]. Recent literature has revealed many biomarkers that might be useful as a forecaster to predict post-CABG surgery atrial fibrillation [11,12]. The relationship of biomarkers that show tangible confirmation supporting clinical outcome has significantly advanced the field of medicine, helping clinicians in many medicine sub-specialties to forecast clinical course.

The potential for micro-RNAs (miRNAs) to evaluate cardiovascular disease as a non-invasive molecular biomarker is of increasing interest due to their abundant presence in in serum, plasma and urine [13,14,15]. Recently published literature showed that miRNAs that regulate gene expression are involved in the arrhythmogenic substrate of AF [16,17,18]. Further, experimental evidences reported the specific role of miRNAs in defining development or onset of arrhythmia and other cardiovascular disorders [18,19,20,21,22,23,24,25,26,27]. Several techniques have been established to compute miRNAs such as droplet digital polymerase chain reaction (PCR), quantitative stem-loop RT-PCR, chip-based digital PCR, quantitative real-time PCR (qRT-PCR) as well as RNAseq and microarrays [28,29,30].

Along with miRNAs, circular-RNAs (circRNAs) are greatly stable due to a resistance to their exonucleases and debranching enzymes [31,32]. Therefore, circRNAs hold distinctive benefits and may also be beneficial to identify a group of CABG patients who are at risk of POAF [11].

Besides miRNAs and circRNAs, the genomic biomarkers have also shed light on the molecular mechanisms that lead to structural and conductive atrial remodeling, creating an arrhythmogenic substrate for AF development [33]. Thus, the patient-specific genomic sequence may assist in finding the degree to which differentially expressed genes in the atrial tissue samples are linked with an increased risk for POAF in patients undergoing CABG.

To date, the relationship between the function of miRNAs, circRNAs, and gene expressions with POAF risk development in CABG patients has not been thoroughly reviewed in published literature. In this narrative review, we report the available experimental evidence of several miRNAs such as miRNA-483–5p [34], miRNA-29a [35], miRNA-23a [36], miRNA-26a [36], miRNA-199a [16], miRNA-1 [37], and miRNA-133a [37], one circRNA: circRNA-025016 [38], and selected gene expressions such as mitochondrial DNA (mtDNA) [39,40], and other single nucleotide polymorphisms (SNPs) such as vesicular overexpressed in cancer–prosurvival protein 1 gene (VOPP1) [41], rs3740563 [42], rs10504554 [43], rs2249825 [44], rs4572292 [45], rs11198893 [45], rs10033464 [46,47], rs2200733 [46,47,48] and rs13143308 [48] used previously as potential elements predicting POAF risk following CABG surgery.

## 2. Materials and Methods

### 2.1. Design of Study

We intended to assess relevant studies by examining the quality of the previously reported role of miRNA, circRNA and SNPs collected from either tissue, blood or plasma preoperatively and perioperatively among patients undergoing CABG surgery with and (or) without cardiopulmonary bypass (CPB). Articles were extracted using both PubMed and MEDLINE databases. The search strategy involved the MEsH keywords such as “atrial fibrillation”, “Coronary Artery Bypass”, “miRNAs”, “circRNAs”, “mtDNA”, “SNP(s)”, and the text keywords such as “postoperative atrial fibrillation” and “Coronary Artery Bypass Graft”.

### 2.2. Data Extraction

**Figure 1:**
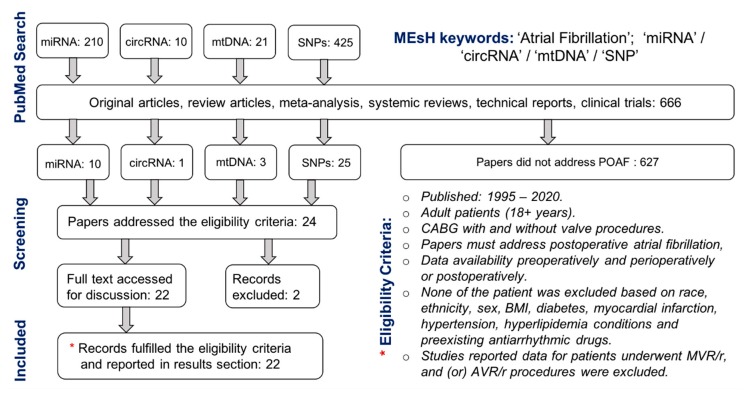
Flow diagram of selected studies searched and reported in this narrative review.

An abstract’s general information for each paper was assessed and studied to ensure both inclusion and exclusion criteria. Studies not published as full-text articles such as published abstracts, single case reports, opinion articles, editorial letters and articles not written in English were excluded. No article was excluded based on pre-existing antiarrhythmic drug therapy. Patient’s w/wo AF history were included. Both prospective and retrospective studies were also included. Search was restricted to studies in adults (aged: 18 + years) w/wo POAF incidence after surgery but none of the studies were excluded based on sex, race/ethnicity, BMI, obesity, diabetes mellitus and myocardial infarction condition. This narrative review is focused on only CABG patients. However, to increase the number of studies and patient population, along with CABG, patients underwent CABG (with or without valve surgery procedures) were also considered and included in this narrative review. Studies reported data for patients underwent only valve surgery such as mitral valve replacement/repair (MVR/r) and aortic valve replacement/repair (AVR/r) were not included. Finally, studies reported the postoperative data for miRNA, circRNA, and gene expressions were included as long as their results exhibited preoperative data for the same parameters. Since this narrative review was focused on cardiac patients’ samples (blood, serum and tissue) collected preoperatively and (or) intraoperatively, the previously reported in vivo studies investigating miRNA, circRNA, mtDNA and SNPs in small and large animal models were also excluded. Summaries of the clinical articles’ selection, data extraction and evaluation are shown in Figure 1.

## 3. Results and Discussion

### 3.1. Micro-RNAs Predicting POAF

There are numerous studies reported that AF is associated with altered miRNA levels in atrial tissue and plasma [20,49,50,51]. miRNAs targeting pathways associated with the regulation of cardiomyocyte metabolism (miRNA-208a and miRNA-223) may alter the metabolic energy reserve required to maintain AF [23,52,53], whereas other miRNAs are thought to play a dominant role in changes related with structural (miRNA-133, miRNA-590, miRNA-29b, miRNA-208, miRNA-638, and miRNA-150) and electrical remodeling (miRNA-328, miRNA−1 and miRNA−26) [54]. Further miRNA−328 [55] and miRNA−29 [56] have also been demonstrated to be potential contributors in AF. Though numerous clinical studies have been reported, the detailed underlying mechanism of onset and persistence of POAF has not been completely elucidated. Mariscalco et al. explained numerous factors which could contribute to POAF risk development following CABG surgery such as atrial dilation, loss of connexins, autonomic imbalance, trauma, ischemia, mechanical myopericarditis, sutures, inflammation, and dysfunction caused by post- extra-corporeal circulation [57]. Similarly, Jalife & Kaur and Santulli et al. focused on a promising contribution of miRNA in similar circumstances [27,58,59]. On the contrary, Krogstad et al. studied plasma collected from 92 CABG patients, reported over 105 miRNAs [60]. In their work, 27 patients (29.4%) developed POAF, and interestingly, they did not find any single miRNA linked with the POAF onset. In the following sub-sections, we report selected miRNAs that have shown potential in predicting the POAF following CABG surgery.

#### 3.1.1. miRNA−483−5p

miRNA−483−5p is a 22-nucleotide (AAGACGGGAGGAAA GAAGGGAG) intronic mature microRNA which is transcribed with its host gene, IGF2, located on chromosome 11p15.5 [61]. As reported that it has been isolated in several human samples such as brain tissue, myocardium, blood serum and hepatic [61,62]. At present, miRNA−483−5p remains relatively poorly examined. Harling et al. conducted a prospective study comprised of 34 patients undergoing non-emergent, on-pump CABG surgery at Imperial College Healthcare NHS Trust (London, UK), and evaluated the role of circulating miRNA−483−5p [34]. All these patients had no prior history of AF. They collected plasma samples at 24 h preoperatively and at day 2 and 4 postoperatively. Among 34 patients, 13 patients (38.2%) developed the POAF condition. These POAF patients tended to be older (64.4 ± 11.3 years), with a higher percentage being male (69.2%). After standard procedures and miRNAs isolation, they found sixteen miRNAs in POAF patients’ atrial myocardium when compared with those maintaining SR. Specifically, miRNA−483−5p showed a 1.804-fold increase and was overexpressed in the preoperative serum samples. In comparison to preoperative samples, there was a substantial increase in the expression of miRNA−483−5p at 48 h time point in POAF-group, *p* = 0.046; however, in no-POAF group, there was no significant change in serum expression in samples collected preoperatively and 2-day postoperatively. Interestingly, both groups exhibited a major increase in miRNA−483−5p expression between 2- and 4- day postoperative time points (POAF group: *p* = 0.0051; no-POAF-group: *p* = 0.0055). In their study, the mean time to onset of AF was 2.5 days. In their findings, they further emphasized that the exact mechanistic role of miRNA−483−5p requires further examination with evaluation of its host gene transcription and protein expression. Thus, a large patient cohort is required to further examine the individual role of miRNA−483−5p as a potential biomarker for POAF risk prediction among CABG patients with no prior AF history.

#### 3.1.2. miRNA−29a

miRNA−29 family targets a cadre of mRNAs that encode proteins involved in fibrosis, including multiple collagens, fibrillin, and elastin [63]. Thus, down-regulation of miRNA−29 would be predicted to derepress the expression of these mRNAs and enhance the fibrotic response. To explore the potential of miRNA−29a and its association in predicting POAF among CABG patients, recently Rizvi et al., conducted a study with 90 patients with no prior history of AF at Advocate Aurora Research Institute (Milwaukee, WI, USA) [35]. They collected fasted blood samples preoperatively in the morning of the cardiac surgery day. Thirty-four (37.8%) patients with average age of 72.04 ± 10.7 years developed POAF. In their findings, they did not report any significant difference in patients’ baseline comorbidities. They further did not observe any significant differences in other risk factors such as diabetes, previous heart attack, high blood pressure, sleep apnea and stroke in patients who developed POAF compared with those who remained in sinus after cardiac surgery. Preoperative amino-terminal-procollagen-III-peptide (PIIINP) and carboxy-terminal-procollagen-I-peptide levels were low in group of patients that remained in sinus after cardiac surgery in comparison to those who developed POAAF with a decline in miRNA−29a. Therefore, this is the first prospective study exhibiting the role of miRNA−29a in association with POAF. Thus, combining age as the only significant clinical predictor with PIIINP and miRNA−29a provided a model that identified POAF patients with higher predictive accuracy. However, this study is limited to only 90 patients and thus, it does not allow to propose miRNA−29a that may be of high clinical relevance in predicting POAF risk development independently. Thus, a larger study is needed to confirm the diagnostic capacity of miRNA−29a in CABG patients with no prior history of AF.

#### 3.1.3. miRNA−23a and miRNA−26a

miRNA−23a is a muscle specific miRNA and is richly expressed in myocardial cells [64]. It was revealed as a novel potential biomarker for diagnosing acute aortic dissection [65]. Similarly, Jansen et al. described the kinetics of another miRNA (miRNA−26a) to be involved in various cardiovascular pathologies [66]. The expression of miRNA−26a was noted to be significantly reduced in atrial samples collected from patients and large animals (dogs) with AF as compared to without AF (control group) [67]. Thus, to further explore the key role of miRNA−26a and miRNA−23a) in predicting POAF risk development, Feldman et al. reported a study to identify patients who developed POAF after undergoing CABG surgery and compared circulating blood levels of miRNA−23a and miRNA−26a between two groups (POAF, *n* = 24 vs. no-POAF, *n* = 24) at preoperative and postoperative time points [36]. They harvested peripheral venous blood preoperatively and 48 h after CABG surgery. The results revealed that the expression levels for miRNA−23a (*p* = 0.02) and −26a (*p* = 0.01) in the POAF group were reduced during the postoperative period in comparison to preoperative results with receiver operating curve of 0.63 (confidence interval [CI]: 0.51–0.74) and 0.66 (95% CI: 0.55–0.77), respectively. However, we envisage that a large prospective study assessing preoperative miRNA−23a and −26a in classifying patients’ POAF risk is therefore essential before they can be recognized as potent biomarkers of predicting a high risk of POAF.

#### 3.1.4. miRNA−199a

The cardiomyocyte-specific microRNA, miRNA−199a, is primary involved in the regulation of (Sirtuin1) SIRT1 expression in cardiac tissue [68]. SIRT1 is a cardioprotective protein involved in the regulation of angiogenesis, prevention of endothelial dysfunction, and counteraction of deleterious effects of ischemia reperfusion injury [68,69,70]. The level of miRNA−199a is lowered with cardiac ischemia, and thus allows an increase in SIRT1 in cardiomyocytes [71]. An enhanced SIRT1 expression is associated with the occurrence of AF [72,73,74]. Yamac et al. reported that an expression of miRNA−199a in 49 patients undergoing CABG procedure. Samples were collected from right atrial appendage tissue and miRNA−199a was lowered in 29 patients that developed POAF after surgery in comparison to 20 patients that remained in sinus (*p* = 0.022) [16]. Since, miRNA−199a was drastically downregulated in tissue probes of patients suffering from POAF, SIRT1 protein was significantly upregulated in tissue probes of patients with POAF (*p* < 0.001). This was the only study that reported the miRNA−199a of patients undergoing CABG surgery. Further work is warranted to develop a multicenter study comprised of large cohort of patients for reproducibility, and its clinical applicability at large.

#### 3.1.5. miRNA−1 and miRNA−133a

Both miRNA−1 and miRNA−133 are the most abundant miRNAs in the heart as they are expressed from bicistronic transcripts containing miRNA clusters [75]. Specifically, patients with persistent AF, miRNA−1 is downregulated in comparison with patients that remain in sinus [76]. Tsoporsis et al. conducted a small prospective patient study (*n* = 42) and collected right atrial appendage samples and venous blood pre- and post-CABG to evaluate the effectiveness of miRNA−1 and −133 [37]. In comparison to patients (*n* = 24, 77.7%) who remained in sinus after cardiac surgery, the group of patients who developed POAF expressed no differences in pre and post CABG levels for both miRNA−1 and miRNA−1. Similarly, in their findings, they did not observe any statistically significant differences in plasma samples collected pre- and post CABG in either of the group (POAF vs. no-POAF). All of the consented patients had no preoperative AF history.

### 3.2. circRNA Predicting POAF

circRNAs have distinctive advantages in comparison to miRNAs in identifying POAF risk among cardiac surgery patients with no preoperative history of AF [11]. Zhang et al. demonstrated a retrospective study with 13,617 plasma circRNAs expression profiles in group of patients that developed POAF and those who remained in sinus after cardiac procedures [38]. Interestingly, their selected circRNAs that were associated with POAF risk development were further validated in two separate and independent cohorts of patients who underwent isolated off-pump CABG. In their work, specifically, an independent cohort of 284 patients (CABG surgery with no prior history of AF) was included to investigate the functioning of the specific circRNA i.e., circRNA_025016). After filtering 31 circRNAs, only nine of them revealed a fold change of more than four in patients who developed POAF as compared to those who remained in sinus. All analyses were conducted via standard qPCR. With further analysis of patient plasma samples, circRNA_025016 revealed the strongest linked with POAF risk and was also found to be elevated in all CABG patients in comparison with healthy controls. Nevertheless, these findings should be further assessed in larger prospective multicenter studies to clarify its role in predicting POAF risk development.

### 3.3. Gene Expressions Predicting POAF

To investigate electrical and structural atrial remodeling, genetic association could be helpful in defining the molecular mechanisms in creating a substrate for AF [33]. The gene expression pattern in atrial tissue might be useful in determining the extent to which the differentially expressed gene(s) in the human atrium are linked with a high POAF risk in CABG surgery patients.

#### 3.3.1. Mitochondrial DNA (mtDNA)

In peripheral blood, mtDNA is found to be linked with a patient’s oxidative stress [77] and is traced in close vicinity of the main cellular source of reactive oxygen species [78]. It has also been reported that oxidative stress plays a critical role in post-surgery AF development in cardiac surgery patients [11,79,80,81]. Zhang et al. measured mtDNA retrospectively using the standard qRT-PCR in peripheral blood collected preoperatively from 485 CABG patients without prior history of AF [39]. The mtDNA copy number was drastically higher in patients with POAF (*n* = 101, 21%) than in those who remained in sinus following CABG procedure (*p* < 0.001). They further investigated that age was not a critical parameter for POAF development in their study. This may imply that mtDNA copy number can be an independent preoperative biomarker for POAF risk. The presented results in their study highly indicate that patients with increase mtDNA copy number may be prone to POAF after cardiac surgery. In another study reported by Sandler et. al., [40], mtDNA was investigated at three different time points (preoperatively, after CPB within 90 min of decannulation, and postoperatively at day 1 and day 2) from 16 patients enrolled prospectively. In comparison to preoperative results, mtDNA in their blood samples was significantly elevated following CPB (six-fold increase post-CPB, *p* = 0.008 and five-fold increase 1–2 days postoperatively, *p* = 0.02). Patients with POAF showed an increase in mtDNA post-CPB than those with no-POAF. Further, patients who developed POAF exhibited at least a two-fold increase of mtDNA postoperatively, whereas this happened in less than 50% of patients without POAF (*p* = 0.037). Their results indicated that the tissue damage and the relevant inflammation initiated by surgery on CPB play a critical function in POAF development, and thus, this confirms that there may be a mechanistic existence at molecular level between mtDNA and POAF. Consequently, future studies are required to assess oxidative stress that impacts mtDNA copy number in the development of POAF risk among CABG patients with no prior history of AF.

#### 3.3.2. Single Nucleotide Polymorphisms (SNPs)

Genetic variation in the G protein-coupled receptor kinase 5 genes (GRK5) potentially acts as a physiological regulator of β-adrenergic receptor activity [82,83,84,85] and is associated with POAF development in CABG (on-pump) patients that are treated with β-blockers (BBs) perioperatively [42]. BBs are used to prevent post-surgery AF developments and as the treatment as well; however, about 20% patients still develop AF following CABG despite having BBs [86]. Kertai et al. noted the same and reported that genetic variation in GRK5 is strongly linked with POAF despite perioperative BB therapy in patients undergoing CABG surgery [42]. In their study with 245 on-pump CABG patients, they isolated genomic DNA from whole blood using standard procedures at Duke Genomic Analysis Facility and tested 492 SNPs. Of the 492 SNPs examined, three SNPs (rs11198893, rs3740563 and rs10787959), belong to the intragenic region of GRK5, showed an increased risk for POAF in 42 patients (17.1%) despite preoperative BBs therapy. Further, among three SNPs, rs3740563 revealed the most significant marker statistically associated with an increased risk for POAF development. Later the same group extended the study with RAA tissue samples. They analyzed the raw data from gene expression profiling in 45 patients with no prior AF history and underwent on-pump CABG surgery [41]. Among 45 patients, 13 (28.9%) developed POAF in spite of preoperative BB therapy. Finally, to further investigate that how sets of genes might be systematically changed their behavior in patients with POAF in comparison with no-POAF, they demonstrated gene set enrichment scrutiny. The most significant search was vesicular overexpressed in cancer - prosurvival protein 1 gene (VOPP1) which showed 1.83-fold change (*p* < 0.01) and was found to be up-regulated in patients that developed POAF. The second most significant probe was LOC389286 gene which revealed 0.49-fold change (*p* < 0.01) and was found to be down-regulated in patients that developed POAF. These results depict that patients undergoing CABG surgery, RAA gene expression profiling can be helpful scientifically to study VOPP1 as it has a critical role in the development of POAF despite using BBs therapy preoperatively. Therefore, the mechanisms that connect atrial VOPP1 expression with the development of POAF in cardiac surgery patients remains unclear.

Kertai et al. conducted a gene-wide association study in two cohorts of patients (diversity, *n* = 877 and validation, *n* = 304) to investigate the link of a genetic polymorphism in lymphocyte antigen 96 (LY96) with POAF incidence in CABG patients [43]. Based on SNPs selection criteria, they recognized only the minor allele of rs10504554, in the intronic region of the LY96, which exhibited a lower risk for POAF in both data sets (discovery data set: OR 0.48, 95% CI 0.34–0.68, *p* < 0.01, and replication dataset: OR 0.55, 95% CI 0.31–0.99, *p* = 0.046). These evidences in two different groups: discovery and validation, conclude that a SNP (rs10504554) is associated with decreased risk of POAF in patients undergoing CABG surgery. A prospective cohort study with 128 patients was conducted by Qu et al. to study the relationship between rs2249825 (C/G) polymorphism in high-mobility group box protein 1 (HMGB1) and POAF in patients who underwent CABG under CPB [44]. POAF incidence occurred in 37 (28.9%) patients. Blood samples were collected before, and after (at 4, and 24 h) CPB. Enzyme immunoassay was used to quantify HMGB1 level. In their findings, they reported that plasma HMGB1 level was increased 4 h after CPB (*p* < 0.0001) and was still increased at 24 h (*p* < 0.0001) in comparison to HMGB1 levels quantified in pre-CPB blood samples. Several epidemiologic cohorts have shown an association between SNPs in the chromosome 4q25 region and the development of AF [87,88,89,90]. The SNPs in the same region have also been associated with an increased risk of AF recurrence after catheter ablation [89]. Earlier retrospective studies reported that polymorphisms in chromosome 4q25 are associated with the development of POAF [46,48]. 

In the study of 1166 white participants from the TexGen genetic registry conducted by Virani et al., [46] the overall POAF incidence after CABG was 36.45% and variants in 4q25 were associated with an increased risk of POAF. In their findings, both rs2200733 and rs10033464 were associated with POAF (OR 1.41, 95% CI 1.04 to 1.91, and OR 1.47, 95% CI 1.05 to 2.06, respectively). Similarly, in two independently collected cardiac surgery cohorts (discovery, *n* = 959 and validation, *n* = 494) conducted by Body et al., non-coding SNPs within the chromosome 4q25 region were independently associated with POAF following CABG surgery [48]. They prospectively collected genomic data from patients undergoing primary CABG surgery with CPB at three major United States cardiovascular centers. They identified rs2200733 and rs13143308 as two SNPs by deCODE8 in the discovery cohort in POAF group (*n* = 289, 30.1%) and were also validated in the validation cohort (*n* = 151, 30.6%). Both rs2200733 and rs13143308 were significantly associated with POAF (rs2200733, OR = 1.97, 95% CI = 1.24–3.15 and rs13143308, OR = 1.76, 95% CI = 1.2–2.52). On the contrary, based on another prospective study conducted by Sodhi et al. [47] with 160 patients undergoing both on/off-pump CABG surgery, SNP markers (rs2200733 and rs10033464) were not predictors of POAF incidence following cardiac surgery. In their findings, POAF occurred in 16% (23) of the patients. Interestingly, in their quantitative results, 30% of total patients revealed a positive genetic test and these patients did not develop POAF. This reduced the positive predictive value to 8% and a negative predictive value increased to 86%. Therefore, this implies that genetic testing cannot be utilized on an individual level to predict the development of POAF risk. Thus, the reported results in different prospective and retrospective studies require detailed validation through multicenter studies in a large cohort.

## 4. Limitations

In the reported literature, we observed significant inconsistencies between tissue studies and plasma samples that could be the result of the biological variation among the patients enrolled in the respective studies. Most of the presented results were extracted based on small cohort population. We also noted that most of the studies have shown only discovery group and the results were not validated in the independent validation group. Only two studies showed results for both the discovery and the validation groups [48], [43]. Most of the studies have patient population w/wo preexisting AF. Further, it has been observed that miRNA expression can be tissue specific and the expression levels for these tissue samples are highly dependent on their origin which can be right atria (RA) or left atria (LA).

Collecting a tissue sample from RAA has some limitations that could limit the contribution to predicting POAF risk development following CABG surgery. First, the RAA tissue used for gene expression profiling was sampled at the time of venous cannulation before sCPB, but a second RAA tissue sample was not collected aright fter terminating CPB. Thus, possible acute alterations in the pre-existing gene expression patterns that may result from myocardial ischemia/reperfusion injury and has not been studied in some of the reports.

Atrial fibrillation frequently originates from the pulmonary veins in LA, while only a small portion originates from the superior vena cava or the inferior vena cava, or in the RA [91]. Therefore, those studies that collected tissue samples from RAA and exhibited gene expression profiles may not completely reveal the gene expression patterns that could truly contribute to POAF development [92]. There were also studies that reported the results based on tissue sample collected from LA in CABG patients. Nevertheless, this would increase the risk of complications, and these studies may be helpful for research only, but they are not easy to implement clinically.

There are several drugs that could potentially add bias in the results such as the most commonly used one is heparin. It has also been observed that sometimes, the patient blood samples may have been stored in holding tubes containing heparin. It is commonly seen that heparin obstructs the enzymes in the PCR and thus may affect its results. As shown in Table 1, results for sensitivity, specificity and AUC have not been reported, and this limits the repeatability of the given data.

## 5. Future Perspective

This narrative review demonstrated experimental evidence of miRNAs, circRNA and selected gene expressions of mtDNA and SNPs as valuable predictors of POAF risk development in cardiac surgery patients following CABG. Although our knowledge of the roles of mi/circ-RNAs and selected gene expressions has significantly improved, additional research is highly recommended with larger patient cohorts to validate these selected mi/circRNAs and gene expressions as potential biomarkers for diagnosing POAF risk development. Finding appropriate miRNAs in serum/blood samples preoperatively may provide more details on molecular mechanisms that lead to electrical and structural atrial remodeling, revealing which cardiac surgery patients are at greatest risk for POAF development.

Preoperative miRNAs, circRNA and SNPs may also uncover patients that would benefit from increased post-surgical monitoring, pre-emptive antiarrhythmic therapy, and further personalized treatment strategies, such as prophylactic surgical interventions (surgical Maze, surgical pulmonary vein isolation, etc.) to minimizing the risk of developing long-term AF. Future studies may emphasis on improved understanding the multifactorial mechanisms of POAF risk development. This narrative review suggests an important need to focus on the mechanisms of changes in signaling pathways in patients who are at POAF risk. Further, we suggest the following outlines to develop translational clinical research to assess and authenticate the validity of the reported miRNAs, circRNA, mtDNA and SNPs:

- A large cohort study is warranted to investigate multivariate aforementioned parameters (miRNA, circRNA and gene expressions).
- Prospective study should be established with cardiac surgery patients with no preoperative AF history.
- Multicenter studies should enroll cardiac surgery patients regardless of their race, ethnicity, sex, BMI, diabetes, COPD, hypertension, hyperlipidemia, PVD, PAD, myocardial infarction, PCI, TIA and CAD.
- For each patient, pre-, intra- and post-operative antiarrhythmic drug record must be reported.
- Each patient blood sample must be collected preoperatively at two time points (24 h and 6 h) before surgery. RAA tissue sample can also collected intraoperatively.
- Same technique (qPCR, eQTL or OMNI1-Quad BeadChip) must be implemented to test assay.
- Electrophysiology findings such as conduction velocity and refractive period should be co-related with the levels for miRNA, circRNA and SNPs.
- Patients must be categorized as ‘no-POAF’ for those who do not develop post-surgery atrial fibrillation and ‘POAF’ for those who develop post-surgery AF within 1–4 days cardiac surgery.
- Results must be reported for both discovery and validated groups.

## Figures and Tables

**Table 1:** mi/circRNAs and other gene expressions recently reported to be used as predictors of POAF development among CABG patients.

RNAs and Gene Expression	Protein/Gene/Loci	CABG	Source	All Patients	Study Type	With POAF	*p* Value	AUC	Sensitivity	Specificity	Technique	Ref.
miRNA−483−5p		On-pump	Blood	34	Prospective	12 (35.3%)	0.046	0.78	77.78	77.27	qPCR	[34]
miRNA−26a		On-pump	Serum	48	Prospective	24 (50.0%)	0.010	0.66	-	-	qPCR	[36]
miRNA−23a		On-pump	Serum	48	Prospective	24 (50.0%)	0.020	0.63	-	-	qPCR	[36]
miRNA−199a	SIRT1	On-pump	RAA	63	Prospective	20 (31.7%)	0.022	-	-	-	qPCR	[16]
miRNA−1 and miRNA−133a		On/Off-pump	RAA	42	Prospective	14 (33.3%)	<0.05	-	-	-	qPCR	[37]
circRNA−025016		Off-pump	Plasma	284	Prospective	68 (23.9%)	<0.01	-	73.52	77.83	qPCR	[38]
mtDNA		Off-pump	Blood	485	Prospective	101 (20.8%)	<0.01	0.81	70.3	80.2	qPCR	[39]
mtDNA		On-pump	Plasma	16	Prospective	6 (37.5%)	<0.01	-	-	-	qPCR	[40]
SNP (VOPP1)		Off-pump	RAA	45	Prospective	13 (28.9%)	<0.01	-	-	-	eQTL	[41]
SNP (rs3740563)		On-pump	Blood	245	Prospective	42 (17.1%)	0.011	-	-	-	OMNI1-Quad BeadChip	[42]
SNP (rs2249825)	HMGB1	On-pump	Blood	128	Prospective	37 (29.9%)	<0.001	-	-	-	qPCR	[44]
SNP (rs4572292 and rs11198893)	GRK5	On/Off-pump	Blood	1348	Reterospective	405 (30.0%)	<0.01	-	-	-	qPCR	[45]
SNP (rs2200733 and rs10033464) ^#^	4q25	On/Off-pump	Buccal swabs	143	Prospective	23 (16.1%)	NS	-	16	71	deCODE	[47]
SNP (rs2200733 and rs10033464)	4q25	On/Off-pump	Blood	1166	Reterospective	425 (36.4%)	0.048	-	-	-	qPCR	[46]
SNP (rs2200733 and rs13143308)	4q25	On-pump	Blood	959 * 494 **	Prospective	289 (30.1%) * 151 (30.6%) **	<0.01	0.72	-	-	deCODE	[48]
SNP (rs10504554)	LY96	On-pump	Blood	877 * 304 **	Prospective	257 (29.3%) 84 (27.6%)	<0.01	-	-	-	OMNI1-Quad BeadChip	[43]

mtDNA: mitochondrial DNA; VOPP1: vesicular overexpressed in cancer, prosurvival protein 1; SNP: single-nucleotide polymorphism; qPCR: quantitative polymerase chain reaction; eQTL: expression quantitative trait loci; RAA: right atrial appendage. ^#^ In this work, genetic testing exhibited a low sensitivity and positive predictive value in assessing the risk of developing postoperative AF in an individual patient. * and ** represent discovery and validation groups, respectively. NS represents not significant.
